# Supplementary material for: Are carbon emissions trading and green financial instruments synergistic? -Comprehensive quantitative research based on content analysis
Source: PLoS One. 2024 Mar 7;19(3):e0298601. doi: 10.1371/journal.pone.0298601 (PMC10919601; doi:10.1371/journal.pone.0298601)
Supplement: S1 Data — (ZIP) [file pone.0298601.s001.zip › Data/地级市Z_F工作报告.zip!/╣π╢1⁄2/│▒╓▌╩╨/└┤╫╘╣1⁄2╓┌║┼í╛┬φ┐╦╩2╛▌═°í┐_2012.docx]

各位代表：

　　我代表市人民政府向大会作政府工作报告，请予审议，并请政协各位委员和其他列席人员提出意见。

一、过去五年工作回顾

　　2007年以来，在省委省政府和市委的正确领导下，在市人大和市政协的监督支持下，市政府坚持以科学发展观为统领，围绕“三年打基础、五年大变化、十年大发展”的目标要求，积极应对各种困难和挑战，千方百计促发展、扎扎实实惠民生、常抓不懈保稳定，有力地推动了全市经济社会全面发展，圆满完成了市十三届人大第一次会议确定的五年奋斗目标和第六次会议确定的2011年工作任务。

　　（一）经济综合实力跃上新台阶

　　经济保持较快增长。过去五年，主要经济指标年均保持两位数增长。2011年，预计（下同）实现生产总值645亿元，比上年增长13%，比2006年的320.7亿元翻一番，五年年均（以下简称年均）增长13.2%；人均生产总值2.41万元，比上年增长11.8%，年均增长12%。

　　财税金融发展良好。过去五年，财政收入稳步增长，金融形势保持稳定。去年，地方财政一般预算收入26.9亿元，增长15.7%，年均增长20.3%。各项税收收入65.3亿元，增长19%，比2006年增加近1倍。其中：国税国内税收收入42.5亿元，增长18.3%，年均增长13%；地税税收收入22.8亿元，增长20%，年均增长17%。金融机构本外币存款余额709亿元、贷款余额251亿元，分别比上年末增长9%和14%，年均增长13%和12%。

　　经济内生动力增强。过去五年，全社会固定资产投资逐年增加，投资结构不断优化。去年，全社会固定资产投资200.8亿元，增长12%。其中：工业投资112.2亿元，占固定资产投资的55.9%；62个年度重点项目完成投资77.6亿元，占固定资产投资的38.6%。过去五年，累计完成固定资产投资803.3亿元，其中工业投资432.9亿元。实施重点项目143项，完成投资339.5亿元，占固定资产投资的42.3%。

　　（二）产业发展水平获得新提升

　　技术创新能力增强。过去五年，技术改造和创新步伐加快，科技创新服务平台建设加强。全市实施技术改造项目773个,完成技改投资85.5亿元。实施市级以上科技项目762项，新认定高新技术企业38家，新增省级工程技术研发中心14家、省级以上企业技术中心31家、省级专业镇8个，列入省现代产业500强项目23个、省自主创新100强企业4家、省战略性新兴产业项目2个。潮州陶瓷研究院、韩山师院陶瓷学院建成发挥作用，省市共建陶瓷先进制造业基地初见成效。全市专利申请量和授权量连年稳居全省第10位。

　　工业发展水平提升。陶瓷、服装、食品、不锈钢等优势特色产业不断发展，电子、信息、生物等新兴产业逐步形成，长城集团、雅士利集团相继上市。临港产业建设不断推进，工业适度重型化趋势加强，大唐潮州发电厂装机容量320万千瓦的1－4号机组全部建成投产。去年，全市规模以上工业增加值218亿元，比上年增长20%，年均增长19.3%。规模以上企业达到663家，年产值超亿元企业150家。质量强市战略较好实施，国家陶瓷质检中心和6个省级质检机构建成运营，国家食品软包装产品及设备质检中心获得批准筹建，61个单位主导或参与了49项国家标准、26项行业标准和17项地方标准制修订，建成省级产业集群和特色产业基地11个，荣获国家级区域品牌32项、省级以上名牌名标355项。

　　农业产业化程度提高。耕地保护制度得到落实，粮食种植面积和总产量连年超额完成省下达考核指标。生柑、茶叶、花卉、铁皮石斛等特色产业规模不断扩大，深水网箱养殖等现代渔业健康发展。现代农业园区、标准化示范区等建设成效明显，建成现代标准农田15.8万亩。拥有市级以上农业龙头企业32家、流通组织8家、农民专业合作社153家，带动近10万户农户增产增收。农业技术推广、标准化建设和农产品质量安全工作加强，累计获得无公害产品、绿色食品、有机食品认证164项。去年，全市农业总产值77.9亿元，比上年增长4.5%，年均增长3.9%。

　　文化旅游产业发展加快。文物保护和旅游管理体制创新完善，古城文化旅游区保护建设成效明显，广济桥、牌坊街、许驸马府等一批文物景点修复开放，太平街义兴甲巷入选“中国历史文化名街”。各具特色的景区景点得到保护建设，创建国家4A级景区3个。木雕、潮绣、麦秆画等传统工艺生产逐步迈向产业化，旅游商品市场日趋活跃。旅游宣传推介力度加大，文化旅游、自然生态旅游和乡村旅游不断发展，我市成为省内最受欢迎的旅游目的地之一。去年，全市接待海内外游客418.5万人次，旅游收入63.4亿元，均比上年增长17%，年均增长17.1%和18.2%。

　　（三）贸易招商工作迈出新步伐

　　国内市场有效拓展。市场消费活跃，去年全社会消费品零售总额288.5亿元，比上年增长17.5%，年均增长19%。国内市场开拓力度加大，“广东潮州陶瓷暨特色产品（上海）展销会”、“广东（潮州）赴北京商品大集”等展销活动成效明显，规模以上工业产品内销比重从2006年的61%提高到75%。

　　对外贸易投资稳步发展。在严峻的市场环境下，外贸进出口稳定增长。去年，全市进出口总值40.9亿美元，比上年增长7%，年均增长16.8%。其中，出口总值26.9亿美元，比上年增长15%，年均增长11%。企业对外投资步伐加快，累计设立境外投资企业13家。

　　招商引资成效明显。去年，新批外商投资项目30个，实际利用外资1.26亿美元，比上年增长12%。过去五年，新增利用外资项目150个，实际利用外资5.1亿美元。利用内资取得新突破，广州港集团公司与亚太公司合作开发的亚太通用码头计划总投资75亿元，首期投资8.96亿元的工程即将竣工；我市与中交集团合作的潮州港西澳港区开发项目、中国国电集团公司投资的新能源项目正在开展前期工作。

　　（四）可持续发展能力有新增强

　　基础设施建设扎实推进。过去五年，累计完成基础建设投资266.9亿元。汕梅高速公路潮州段建成通车，省道335线、334线、233线、232线、222线、231线潮州段等得到改造提升，市客运中心枢纽站建设基本完成，厦深铁路潮州段和潮汕火车站、饶平火车站进站公路等建设正在推进。新建改建500千伏、220千伏、110千伏输变电站26座，完成一批电网配套工程建设，电力保障能力全面提高。仙洲岛堤围整治和韩江南北堤达标加固等14宗水利工程全面竣工，江海堤围加固106公里，中小型水库达标加固167宗，沟尾溪、内洋南总干、河内湖三大涝区整治工程全部开工建设，水利防灾减灾工程建设连续5年被评为全省先进单位。

　　产业园区建设步伐加快。省级高新技术产业开发区获得省政府批准设立。深圳（潮州）产业转移工业园被认定为省级示范园，已办理入园手续企业99家、投产52家，径南分园基础建设正在进行。潮州港经济区开发建设逐步推进，进港公路等基础设施建设步伐加快。各县区产业园区规划建设工作积极开展。

　　节能减排工作成效明显。市污水处理厂扩建工程完成，潮安、饶平县城污水处理厂建成投用，桥东污水处理厂建设基本完成，7个镇级人工湿地建成发挥作用，市城区污水处理率达到82.5%。市生活垃圾卫生填埋场一、二期工程建成，市区生活垃圾无害化处理率达到100%。资源综合利用和陶瓷清洁生产等工作扎实推进，陶瓷窑炉节能降耗技术在全省推广应用，节能减排工作超额完成省下达的目标任务。饶平县获得“国家绿色能源县”称号。

　　资源管理利用工作加强。新一轮土地利用总体规划修编完成，新开发补充耕地1.5万亩，基层国土资源所全面建立健全，违法违规用地查处工作扎实有效，土地管理工作通过国家土地例行督察并受到好评。非法采矿和破坏性采矿“双打击”专项行动取得成效。

　　（五）城乡发展面貌呈现新变化

　　城市管理有新加强。《潮州市城市总体规划》、《潮州城区部分用地控制性详细规划》、《历史文化名城保护规划》等一批规划修编完成，规划管理力度加大。“重心下移、属地管理”的城市管理体制初步形成，各项管理工作得到加强。环境卫生“双整治”成效明显，脏乱差现象有所改观，我市在国家城市综合整治定量考核中连续多年居全省前列。城市公交管理体制理顺，运营车辆更新换代，公交事业发展迈上新台阶。

　　城市功能不断完善。金山大桥建成通车，一批城市道路得到改造。桥东水厂、竹竿山水厂扩建工程顺利完成，城市供水能力全面提高。三利溪等排涝系统得到整治，市区排水不畅问题基本解决。市文化艺术中心、市博物馆建成，市图书馆、市档案馆正在建设。园林绿化景观建设扎实推进，城市绿地和休闲设施增加，荣获“国家园林城市”称号。

　　农村面貌继续改变。过去五年，全市新增新农村建设示范点86个，创建林业生态文明村197个。建设改造农村公路1796公里，镇通行政村公路硬底化建设提前一年完成省下达的目标要求，符合通车条件的行政村全部开通客运班车。农村无害化卫生厕所普及率达到88%。15.3万人的农村饮水特别困难和65.6万人的农村饮水安全问题全部解决，饶平沿海六镇饮水解困工程全面完成。

　　生态环境逐步优化。国家环保模范城市创建活动积极开展，韩江、枫江、黄冈河流域综合治理稳步推进，海洋生态建设和环境保护加强，大气污染治理力度加大，生态环境保持稳定。韩江潮州河段环境整治与滨江景观建设，被授予“中国人居环境范例奖”。林业生态工程建设成效明显，建成生物防火林带478.6公里，森林覆盖率达到61.2%，潮安县、饶平县先后通过省林业生态县验收。

　　（六）各项民生工作取得新发展

　　人民生活水平提高。去年，城镇居民人均可支配收入15583元，比上年增长14%，年均增长10.2%；农民人均纯收入7648元，比上年增长20%，年均增长12.1%。去年，全市用于社会民生事业的财政资金38亿元，占地方财政一般预算支出的62%，“十项民心工程”得到有效推进。

　　就业保障工作加强。去年，城镇登记失业率2.21%，低于全省平均水平。过去五年，城镇累计增加就业岗位14.4万个，全社会非农就业比重超过80%。农村劳动力培训和转移步伐加快，累计培训11.1万人、转移就业24.6万人。社会保障体系不断完善，城镇养老、医疗、失业、工伤参保人数达到43.2万、55万、33.7万、32.8万，分别比2006年增长60.6%、1098%、49.8%和53.3%。养老保险待遇大幅度提高，企业退休人员人均每月养老金1087元，比2006年增加674元。4.73万名困难国有集体企业退休人员得到资助纳入医保范围。新型农村合作医疗继续发展，参合率达到99.96%，补助标准大幅度提高。新型农村社会养老保险试点工作扎实开展，参保人数达到50万人。

　　解困工作不断推进。最低生活保障覆盖面扩大，去年低保人数70791人，比2006年增加48933人。住房保障工作扎实有效，过去五年共开工建设保障房4602套，落实租赁补贴1351户。社会慈善活动广泛开展，“春风暖人心”等爱心工程积极实施。对口援建汶川耿达乡工作成效明显，“广东潮州新村”等项目建设全面完成。农村低收入住房困难户住房改造3454户，15个不具备生产生活条件的贫困村庄整体搬迁。扶贫“双到”成效显著，实施扶贫开发项目940个，贫困村贫困户脱贫率达到95%。

　　社会保持和谐稳定。应急管理工作逐步加强，安全生产形势持续稳定。公安“三基”工程建设全面达标，治安防控能力显著提高，违法犯罪活动受到有效打击。各级信访大厅达标建设和综治信访维稳中心建设全面完成，群体性事件得到妥善处置，综治工作连续多年在省考核中获得优秀等次。制假售假、走私贩私等违法活动受到打击，产品质量和食品药品安全工作加强。

　　社会事业协调发展。城乡免费义务教育政策全面落实，职业技术教育加快发展，普高教育发展质量提升，高中阶段毛入学率达到86%。城乡公共文化设施不断完善，乡镇文化站建设全面达标，农家书屋覆盖全部行政村，国家级非物质文化遗产保护项目达到12个。“潮州土楼”成为国家第三次文物普查“十大新发现”之一。市中心医院扩建工程完成，潮安县人民医院建成投用；医疗卫生体制改革扎实推进，基层卫生院全面实施国家基本药物制度，基本医疗保障水平进一步提高。人口计生工作进入省先进行列，两县两区均达到省一类地区标准。群众性体育设施加快普及，全民健身运动广泛开展。外事侨务工作取得新成效，过去五年共接受旅外乡亲捐赠2.65亿元。基层民主政治建设稳步推进，城乡文明建设加强，连续5次荣获“广东省文明城市”称号。行政服务、双拥共建、人民武装、人事编制、审计、统计、人防、粮食、物价、供销、贸促、气象、防震、地方志、档案、老龄、民族宗教、人文社科、妇女儿童、残疾人和红十字等工作取得新发展。

　　在经济社会取得新发展的同时，政府自身建设继续加强。民主法治建设加快，“五五”普法工作全面完成。市县区政府机构改革和第四轮行政审批事项清理调整工作顺利完成，投融资体制、文化体制、集体林权等改革不断深化。依法行政工作继续加强，行政管理和服务水平不断提高，为民服务全程代理制全面推广。政务公开深入开展，电子监察系统逐步健全，政风行风热线节目效果良好。向市人大报告工作、向市政协通报情况制度逐步完善，与各民主党派、人民团体的联系进一步密切。五年来，累计办理省、市人大代表建议235件，市政协委员提案467件。政府廉政建设责任制全面落实，一批违法违纪案件受到严肃查处。

　　各位代表，过去五年，我市能够有效应对各种困难和挑战，较好地保持了经济社会平稳较快发展，主要得益于我们紧紧把握住以下要点：一是坚持解放思想、改革创新。重视把握发展这个第一要务，解放思想、与时俱进，积极推进改革、创新、发展，有效地应对了各种困难和挑战。二是坚持科学发展、加快发展。围绕转变经济发展方式这个中心，大力加强产业建设，努力做大经济总量，着力提升发展质量，积极在发展中打基础增后劲。三是坚持以人为本、服务民生。按照发展为了人民、发展依靠人民、发展成果惠及人民的要求，着力改善民生、服务民生，以实施“十项民心工程”为载体，切实为群众办实事谋福祉，努力推动“幸福潮州”建设。四是坚持依法行政、执政为民。以提高施政能力和服务水平为目标，强化依法行政和执政为民理念，切实转变作风，狠抓责任落实，较好地推动了各项事业发展。

　　各位代表，过去五年取得的成绩，是省委省政府和市委正确领导的结果，是市人大和市政协监督支持的结果，是全市人民共同奋斗、辛勤努力的结果，是驻潮部队、武警官兵、上级驻潮各单位，以及港澳台同胞、海外侨胞和社会各界鼎力相助的结果。在此，我代表市人民政府表示衷心的感谢并致以崇高的敬意！

　　在看到成绩的同时，我们也清醒地认识到，我市经济社会发展仍存在不少困难和问题。主要是：经济总量较小，财政压力较大；产业结构不够合理，创新能力仍需提高；城市化水平相对偏低，发展环境有待改善；人民生活水平不高，改善民生任务较重；资源环境压力加大，社会管理还需加强；办事效率不高，作风建设有待加强。对此，我们要高度重视，积极采取措施加以解决。

二、今后五年的奋斗目标和主要任务

　　今后五年，是我市全面实施“十二五”规划，加快转型升级、建设幸福潮州的关键五年。面对复杂多变的国际政治经济环境和国内经济运行的新情况新变化，市政府将按照中央和省的决策部署，认真贯彻落实省委十届十一次全会和市第十三次党代会精神，团结带领全市人民继续解放思想，创新发展理念，转变发展方式，破解发展难题，提高发展质量，努力开创一个又好又快的发展新时期。

　　根据市十三次党代会提出的目标要求，今后五年我市经济社会发展的主要目标是：全市生产总值年均增长11.5%，人均生产总值年均增长10.5%，地方财政一般预算收入年均增长16%，城镇居民人均可支配收入年均增长10%，农民人均纯收入年均增长12%。做好今后五年的政府工作，要深入贯彻落实科学发展观，以加快转变经济发展方式为主线，以“加快转型升级、建设幸福潮州”为目标，实施创新驱动、产业升级、文化引领、绿色发展、城市拓展、惠民共享六大战略，继续构建国内外有重要影响力的历史文化名城、国内重要的特色产业基地、广东重要的临港产业基地，着力打造“产业优化升级先行市、文化经济融合示范市、社会管理模式创新市、绿色生态环保模范市”，努力把潮州建设成为创新宜业、生态宜游、惠民宜居的幸福名城。重点要抓好以下九个方面工作。

　　第一，着力抓好创新能力提高。坚持把科技进步和创新作为加快转变经济发展方式的核心推动力，加强人才培育引进，集聚创新资源，掌握创新技术，强化创新动力，营造创新环境，促进技术创新能力和产业竞争力“双提升”。

　　第二，着力抓好农业农村工作。坚持立足特色、发挥优势，加大农业投入，创新农业科技，完善经营机制，推进农业特色化、产业化、基地化、标准化，大力发展现代高效农业。加强农村基础建设和公共服务，不断改善农村生产生活条件。

　　第三，着力抓好工业转型升级。坚持集约化发展、特色化推进、信息化提升，促进传统优势产业高端化、品牌化、集群化发展。引导企业以股份制等形式进行整合兼并、做大做强，发展壮大实体经济。大力推进产业园区开发建设，积极承接产业转移，培育发展战略性新兴产业，全面提升工业发展水平。

　　第四，着力抓好港口经济发展。坚持把港口经济作为实现跨越式发展的主攻方向，充分发挥港口资源优势，加强港口港区基础建设，加大招商引资力度，实施大项目带动战略，大力发展能源、石化、船舶修造和现代物流业等临港产业，推动港口经济加快发展。

　　第五，着力抓好文化经济融合。坚持以优秀潮州文化为引领，保护传承优秀传统文化，整合文化旅游资源，做强文化旅游品牌。振兴工艺美术业，培育文化创意业，提升文化产业竞争力。建立健全文化发展创新机制，彰显潮州文化在经济社会各领域中的独特魅力。

　　第六，着力抓好重大项目带动。坚持利用国内外资源，加强投资环境宣传推介，创新招商引资办法，大力引进和实施一批关系全局的重大项目。积极拓宽融资渠道，加大财政统筹和投入力度，全力推进交通、水利、能源等基础设施建设，为经济社会加快发展创造良好的环境。

　　第七，着力抓好城市向东拓展。坚持城乡统筹发展，加快潮州大桥、韩江东西溪大桥和相关路网的规划建设，推动城市向东扩展，促进以韩江东岸和潮州港经济区为重点的滨江、滨海新区建设，努力提高城市化发展水平。力争到2016年全市城镇化率达到67%，城市建成区面积超过70平方公里，人口超过65万人。

　　第八，着力抓好绿色生态建设。坚持绿色生态发展方向，加强环境资源保护利用和节能减排工作，大力发展循环经济，加快建设资源节约型社会和环境友好型城市，力争到2016年全市森林覆盖率、城镇生活垃圾无害化处理率、城镇污水处理率分别达到62.5%、85%和75%，城市人均公园绿地面积达到13平方米。

　　第九，着力抓好民心工程实施。坚持把保障和改善民生作为政府工作的出发点和落脚点，加快发展教育、文化、卫生等各项社会事业，健全社会保障体系，加大扶贫济困力度，提高公共福利水平和居民幸福指数。加强社会建设，创新社会管理，完善社会服务，促进社会公平正义，努力建设“幸福潮州”。

我们相信，经过今后五年的努力，潮州发展将更加科学，城市将更加宜居，生态将更加文明，社会将更加和谐，人民将更加幸福，一定会在科学发展的道路上迈出新的步伐、取得新的成绩！

三、2012年的工作意见

　　2012年，是新一届政府的届首之年。做好今年各项工作，对于实现今后五年工作目标至关重要。综合考虑诸多因素，今年全市经济和社会发展的主要预期目标是：生产总值增长11%，单位生产总值能耗下降3.66%，地方财政一般预算收入增长16%，固定资产投资增长12%，外贸出口增长8%，社会消费品零售总额增长15%，居民消费价格指数涨幅控制在4%以内，人口自然增长率控制在6.9‰以内，城镇登记失业率控制在3%以下，市区居民人均可支配收入增长10%，农村居民人均纯收入增长12%。为实现上述目标，要认真贯彻落实中央经济工作会议精神和省委省政府的决策部署，着力保持经济平稳较快发展、物价总水平基本稳定、社会大局和谐稳定，在转变经济发展方式、深化改革开放、保障和改善民生上取得新成效，以优异成绩迎接党的十八大胜利召开。重点是抓好以下工作：

　　（一）以扩大投资需求为动力，保持经济平稳较快发展

　　全力促进投资增长。积极推进一批关系长远、带动全局的重大项目建设，扩大对新型工业、现代农业、现代服务业，以及基础建设、社会民生等重点领域的投资，努力增强投资对经济的拉动作用。切实协调解决重点项目建设碰到的资金筹措难、征地拆迁难等问题，努力排除影响项目建设的各种障碍，力争年度计划投资86.7亿元的50个重点项目建设顺利推进。拓宽投融资渠道，创新项目合作模式，引导民间资本投向，促进社会投资稳定增长和结构优化。

积极拓展内销市场。继续举办具有特色的产品展销会，积极组织企业参加各类经贸活动，不断提高“潮货”的影响力和知名度。鼓励支持企业通过开设品牌店、专卖店等形式，进一步完善营销网络和销售渠道，努力提高“潮货”的市场占有率。优化城乡商业布局，推进“万村千乡”市场工程，建设特色商业街区。完善商贸流通体系，加快发展连锁经营、物流配送、电子商务，提升流通现代化水平。落实鼓励消费政策措施，加强市场价格监管，发展新型消费业态，全面扩大消费需求。

　　着力抓好外贸出口。坚持重点企业联系制度，大力帮扶出口大户扩大外贸出口。引导企业积极应对贸易摩擦，努力防范外贸风险。组织企业参加广交会、高交会等经贸活动，巩固发展外贸出口市场。打造优势产业出口基地，支持自主品牌、自主营销商品出口，扩大机电和高新技术产品出口。支持企业加快“走出去”步伐，建立境外生产基地、营销服务网络，推动贸易和投资良性互动。加强“大通关”建设，营造外贸发展良好环境，全力促进外经贸转型升级。

　　（二）以产业转型升级为主线，推动工业结构调整优化

　　提升特色产业发展水平。以技术改造和技术创新为重点，支持企业加强技术引进和技术开发，积极实施重大科技项目，推动优势产业迈向更高层次。深入实施质量强市战略，鼓励优势企业参与国家、行业和地方标准的制修订，积极组织企业申报名牌名标。引导企业创新经营理念和发展模式，落实鼓励企业上市的政策措施，加大对上市后备资源的培育力度，推动三环、凯普等企业尽快上市。支持传统优势企业向高新技术企业转型，大力发展电子信息、生物制药、新光源照明等新兴产业，发展壮大庵埠食品包装机械、黄冈水族机电、枫溪陶瓷机械等先进制造业，引导陶瓷卫浴等产业提升层次、做大规模。

　　促进临港产业加快发展。创新完善港口港区管理模式，认真组织实施港口港区各类规划，完成进港公路、亚太通用码头等基础设施建设，加快西澳港区综合开发和公用航道疏浚，争取大潮高速公路、疏港铁路、潮州港供水工程纳入规划建设，完成临港产业转移园首期2745亩土地的征收储备，打造良好的投资发展载体。加大招商引资力度，积极引进能源、石化等重大产业项目，配合开展大唐发电厂5、6号机组前期工作，大力推进国电、华能风电场等项目建设，加快实施华丰二期工程、粤东水产品物流中心、饶平韩钢、和洋船舶配套产品基地等项目，促进临港产业发展壮大。尽快启动潮州港对台直航，积极申报设立海峡两岸商品交易物流中心，努力提高对台经贸合作水平。

　　加强产业发展平台建设。深化产学研合作，支持企业设立工程研发中心、技术中心和实验室，引导创新要素向企业集聚。完善潮州陶瓷研究院建设，推动专业镇转型升级，充分发挥陶瓷、食品、不锈钢、水族器材等质检机构的作用，加快建设食品软包装产品及设备国家质检中心，增强产业创新服务能力。推进省级高新区建设，积极引进高新科技项目，推动高新技术产业发展。完善园区公共配套服务设施，抓好产业招商和服务工作，做大园区经济规模。加快深圳（潮州）产业转移园径南分园建设，确保今年有20家以上企业进园建设投产。推进潮安特色产业基地等园区建设，不断优化产业空间布局。

　　加快发展生产性服务业。加强市场流通体系建设，重点推进潮州陶瓷国际交易中心、中国瓷都广东国际陶瓷采购中心、潮汕火车站区商贸物流中心、工业原材料批发市场建设，积极规划建设一批特色产业专业市场，努力解决有产业无市场的问题。依托亚太通用码头、大唐煤码头和小红山杂货码头，大力发展港口物流业。培育壮大金融、信息、工业设计和科技服务等生产性服务业，促进服务业与制造业融合发展。

　　（三）以增加农民收入为核心，提升“三农”发展水平

　　大力发展特色农业。认真落实耕地保护责任制，抓好现代标准农田建设。加强高标准生产基地和产业园区建设，推进农业基地化、标准化发展，做大“凤凰单丛茶”、“岭头单丛茶”、“潮州柑”、铁皮石斛等优势特色农业。调整优化海洋养殖布局，扩大深水网箱养殖规模，促进现代渔业发展。

　　提升农业产业化水平。支持农业龙头企业发展，积极发展农民专业合作组织，完善龙头企业与农户利益联结机制，大力推进农业产业化经营，加强产前、产中、产后服务，促进农业增产增效。加快农业技术创新和推广，提高现代农业技术装备水平。加强农产品品牌建设和质量认证，确保农产品质量安全。

　　推进农业农村基础建设。完善农村水利、道路、客运等基础设施。抓好防洪薄弱环节整治，加大沿海堤围、大中型水闸、小水库达标加固和中小河流、易灾地区生态环境综合治理力度，抓好中小型灌区续建配套等建设，加快实施沟尾溪、内洋南总干、河内湖等涝区整治工程。积极启动农田基本建设示范县申报工作。加强生态宜居名村名镇建设和村庄整治，不断改善农村人居环境。

　　多渠道增加农民收入。全面落实强农惠农政策，努力增加农民转移性收入；鼓励农民优化种养结构，增加生产性收入；加快农村二三产业发展，增加经营性收入；加强农民职业培训和就业服务，增加工资性收入。加大扶贫开发力度，推动贫困户脱贫致富，全面完成“双到”工作任务。

　　（四）以文化资源为依托，促进文化与经济融合

　　打造古城旅游品牌。深化潮州文化研究，挖掘古城文化丰富内涵，重视古城区的保护利用，整合古城文化旅游资源，加强对潮州古城旅游品牌的策划，全面提升古城文化的影响力，逐步把潮州古城打造成为国内外游客向往的旅游目的地。

加强景区景点建设。继续完善广济桥、牌坊街配套设施，做好笔架山潮州窑遗址公园规划，加快韩文公祠橡木园建设，启动己略黄公祠等维修工程，不断丰富古城文化旅游内涵。加快发展环古城生态休闲旅游集群，提升凤凰山、绿岛山庄、紫莲山庄、绿太阳山庄等生态景区景点的发展层次，创建一批3A级以上景区，支持永生源等中医药文化养生旅游示范基地建设。积极发展旅游新门类，增强旅游的参与性和娱乐性。

　　配套完善旅游设施。加强旅游吃、住、行、游、购、娱等配套建设，完善旅游服务功能，改善旅游接待条件，加快旅游指引标识、停车场所等建设，大力发展民居客栈，推动国际大酒店等高星级酒店尽快建成投用。抓好“潮州十大名菜、十大名小食”评选，打造广东（潮州）旅游商品基地，培育发展旅游制造业。精心设计观光、休闲、娱乐和购物一体化的旅游线路，完善旅游优惠套票，努力实现旅游经济效益最大化。

　　加大宣传推介力度。加强旅游整体形象策划，创新旅游宣传推介方式，努力打造潮州文化旅游品牌。注重发挥星级酒店、旅行社、导游在旅游促销中的作用，全面提升宣传促销效果。加强区域旅游宣传促销合作，积极组织“潮州行”系列推介活动。加强旅游从业人员培训管理，规范旅游市场秩序，营造良好的旅游发展环境。

促进文化产业发展。发挥“中国工艺美术之都”的品牌效应，推进潮绣、木雕、麦秆画、手拉壶、大吴泥塑等传统工艺产业化、规模化发展。扶持文化创意产业发展，加快中国瓷都文化创意产业园建设，促进文化与产业融合，实现创意产业化、产业创意化。积极培育音像印刷、演艺演出等文化产业，壮大文化产业规模。

　　（五）以环境建设为保障，增强可持续发展能力

　　强化交通能源支撑。按照“外抓大联网、内抓大配套”的总体思路，大力推进交通、能源、通信等基础建设。狠抓对外路网对接，推进厦深铁路、潮揭高速公路、潮惠高速公路潮州段、潮汕火车站进站公路建设，完成砲浮线等道路改造，改善道路交通环境。统筹优化电网结构，推进城乡电网改造升级，实施220千伏线路工程和110千伏输变电工程及电力配网工程等项目，提升电力保障水平。加快“数字潮州”建设，运用现代信息技术服务发展。

切实抓好节能减排。严格执行投资项目节能评估和环评制度，抓好高耗能行业的节能监管和重点领域节能工作，合理控制能源消费总量。加大落后产能淘汰力度，发展应用低碳清洁能源，大力发展循环经济，以结构调整促进节能减排。加强污水处理设施建设，推行排污许可证制度，控制主要污染物新增排放量。加大执法力度，整治环境污染突出问题。加强机动车排气管理，禁止排气超标车辆上路行驶。

　　加强生态环境建设。大力推进国家环保模范城市创建工作，加快饶平大湖山等生活垃圾处理设施建设，构建生态宜居的城市环境。推进“碧水工程”，加强韩江、黄冈河等饮用水源水质保护，改善枫江水质，构建人水和谐的生态系统。加强海洋、湿地、林地和地质环境保护，推进生态公益林、沿海防护林和生态景观林带等生态工程建设。继续推进农村生产生活环境综合治理，加强农业污染防治和畜禽养殖、水产养殖污染防治，构建优美整洁的农村环境。

推进节约集约用地。实行最严格的节约集约用地制度，认真规范建设用地供给及供后管理，遏制盲目圈地和低水平建设，切实保障重点项目、民生工程等用地需求。探索创新土地管理储备模式，增强政府对土地市场的宏观调控能力。加大力度推进“三旧”改造，确保在今年底前完成历史遗留用地手续完善工作，全面推进试点项目的改造，切实改善城乡发展环境。落实土地管理共同责任制，坚决打击违法违规用地、占地行为。

　　规范矿产资源管理。做好矿产资源开发利用规划编制工作，科学规划采石采砂场所布局，有序投放采矿权和采砂权。整顿矿产资源开发秩序，落实动态巡查责任，坚决打击非法开采稀土、瓷土和河砂等行为。加强地质勘查工作，提高地质灾害防治、监测和预警能力。

　　（六）以统筹城乡发展为目标，加快城市化发展进程

　　加快城市向东扩展。坚持以规划为引领，努力拓展城市发展空间，动工建设潮州大桥、韩江东西溪大桥，畅通韩江东西两岸的城市连接。加强韩东片区城市路网等基础设施规划建设，抓好潮州东大道、体育会展中心的规划设计，高起点高标准谋划一批重点基础设施和公共服务设施项目，带动中心城区跨江向东发展。

加强城市基础建设。坚持扩大规模与完善功能并举，加快城市基础设施建设，完善城市路网格局，推进外环路、枫韩线市区段建设，启动城新西路、彩虹路改造工程，提高城市道路畅通水平。加强园林绿化美化工作，抓好排污渠道箱涵建设和路灯线路改造，实施城市垃圾分类处理，巩固提升园林城市建设水平。重视城市公共服务设施建设，配套完善金融、教育、文化、医疗、商贸、公共绿地、运动场所和娱乐等设施，方便群众生产生活。

　　完善城市管理机制。按照“重心下移”的要求，落实市、区城管部门管理责任，形成纵管到底、横管到边、全方位覆盖的管理责任网络。加大城市建设执法督察力度，依法拆除违法建筑、违规广告设施，坚决遏制违法违章建设蔓延势头。加强城市交通管理和环境卫生整治工作，着力解决城市“脏、乱、差”等突出问题。加强市民公共道德教育，规范城市公共秩序，提升城市文明程度。

　　统筹城乡区域发展。加快中心城区、城市新区、产业集聚区、县城区和重点镇建设步伐，增强城镇辐射带动功能，培育新的经济增长板块。以推进农业产业集中发展、农民集中居住、土地集中经营为重点，加快建设农村新社区。抓好农村劳动力转移就业, 落实农民工进城入户积分制，促进人口向城镇有序转移。

　　（七）以深化改革开放为引领，增创经济发展新优势

　　推进重点领域改革。深化行政审批制度改革，优化工程建设等审批流程，提高行政审批效能。创新政府公共服务提供方式，推进政府购买公共服务，提高政府公共服务能力。推进事业单位分类改革和人事制度改革，加快国企改革和股份制改造。深化富县强镇事权改革，增强县镇发展活力。

　　提升开放合作水平。加强城市形象和投资环境宣传推介，依托港口资源、文化底蕴深厚等优势，积极开展招商引资和招商选资工作。强化与国内外大型企业和战略投资者的合作，精选以临港产业、高新技术和新兴产业为重点的招商项目，力争在重大项目招商引资上实现新突破。实施乡贤回归“反哺工程”，鼓励吸引旅外乡亲回潮州投资创业。加强与珠三角地区合作，积极融入海西经济区，主动承接发达地区产业转移。推进潮台经贸合作和文化交流，强化与港澳互利合作，促进汕潮揭同城化发展。

　　加快财税金融发展。坚持依法治税理财，创新财税征管服务方式。加强税源管理，拓宽税收渠道，增加税收收入。推进综合治税，强化征管刚性，公平企业税负。完善财政管理体制，优化财政支出结构，扩大公共财政覆盖范围，增加公共服务支出。引入市场竞争机制和资本运营理念，整合盘活政府有形资产和无形资产。加快投融资体制改革，发展小额贷款公司和融资性担保机构。支持金融部门优化信贷结构，强化金融监管，提升金融服务水平。

　　（八）以改善民生为重点，发展各项社会事业

　　千方百计扩大就业。实施积极就业政策，完善创业工作体系，推动创业带动就业。做好高校毕业生、农村富余劳动力、城镇就业困难人员和新成长劳动力的就业再就业工作。加强职业技能培训，提高劳动者就业能力。落实工资集体协商制度，构建和谐劳动关系。力争城镇新增就业3万人，城镇登记失业率控制在3%以内，组织农村劳动力技能培训2万人、转移就业4.5万人。

　　提高社会保障水平。加大社保扩面征缴力度，努力实现应保尽保。健全城乡一体化社会保障体系，启动城镇居民社会养老保险试点工作，完善新型农村社会养老保险制度。推进城乡医疗保障一体化，狠抓医保扩面征缴，增强医保基金筹资能力和抗风险能力。提升社会救助水平，提高低保补助标准。加强社会福利工作，大力发展慈善事业。

　　坚持教育优先发展。完善学前教育体制机制，整合学前教育资源，提高学前教育普及和规范化水平。调整优化中小学布局，撤并一批“麻雀学校”，扩大优质学校办学规模，促进义务教育均衡发展。加快建设义务教育规范化学校，全面完成校舍安全工程建设。巩固提高普及高中阶段教育成果，大力发展职业教育,力争高中阶段毛入学率达到87%。加强师德师风建设，提高教师待遇水平，促进教育教学质量提升。规范招生行为，维护教育公平。

　　加快发展文化事业。加强社会主义核心价值观教育，大力弘扬潮州人勤奋努力、感恩奉献的精神。加快市图书馆、档案馆等重点文化项目建设，完善县（区）、镇（街道）公共文化设施，新建改建一批文化馆、图书馆和村级文化室，积极推进广播电视村村通、农村电影放映、文化信息资源共享等文化惠民工程，切实提高城乡公共文化服务水平。建设有线数字广播电视网络、高清互动数字电视工程。做好潮州文化生态保护区申报工作，实施文化遗产保护工程。

　　增强卫生服务能力。深化医药卫生体制改革，加强医疗卫生服务体系建设，促进公共卫生服务均等化。加快潮州医院、市妇幼保健院、市精神病院扩建工程建设，全力把市中心医院创建成为“三甲”医院。继续实施卫生信息化工程，提高城乡居民健康档案建档率。加强疾病预防控制，防止重大传染性疾病发生流行。大力开展群众性爱国卫生运动，扎实推进农村改水改厕。广泛开展全民健身运动，增强城乡居民身体素质。

　　加强人口计生工作。强化层级动态管理责任，扎实抓好生育节育政策落实，继续稳定低生育水平。加快基层服务网络规范化、标准化建设，加强流动人口服务管理，完善人口计生利益导向机制，争创省人口计生综合改革示范市。

切实办好民生实事。健全住房保障体系，完成省下达的保障性住房建设任务，改造农村低收入家庭危房2995户。大力实施农村饮水保障工程，建设10.7万人饮水安全工程，启动村村通自来水工程建设。启动“引韩入饶供水工程”的立项申报工作。加快农村道路改造建设，积极发展城乡公交客运，改善群众出行条件，新建县乡公路40公里、通自然村公路200公里，城市公交运营车辆增至151辆、线路增至18条。

　　推进平安潮州建设。健全应急管理体系，提高预防和应对公共突发事件能力。完善社会治安防控体系，整治社会治安重点地区和突出问题，依法打击各类违法犯罪活动。重视信访、维稳和人民调解工作，全力维护社会和谐稳定。落实“六五”普法规划，推进依法治市进程，构建社会主义法治社会。强化安全生产监督管理，防范和遏制重特大事故发生。按照省的部署认真开展“三打两建”活动，加大打假治劣力度，抓好食品、药品、保健品、医疗器械等安全监管，确保人民群众饮食和用药安全。

　　统筹做好其他工作。继续做好人才、民族宗教、新闻出版、对台、人防、气象、防震、统计、档案、地方志、妇女儿童、老龄、残疾人和红十字等工作，促进各项事业全面发展。

　　（九）以推进管理创新为抓手，加强政府自身建设

　　建设创新型政府。坚持解放思想、不断学习、与时俱进，用发展的眼光和科学的态度，研究解决问题、思考谋划未来，以创新的精神和务实的作风，全力推动政府工作上新水平，更好地为改革发展大局服务，为广大人民群众服务。

建设法治型政府。坚持依法行政，自觉接受市人大和市政协的监督，认真负责办理人大代表建议和政协委员提案。广泛听取各民主党派、工商联、无党派人士意见，发挥工会、共青团和妇联等人民团体作用。深入推进政务公开，健全行政决策程序，规范行政行为，完善行政执法体制机制，不断提高依法依规办事水平。

建设高效型政府。坚持求真务实，围绕保民生、促发展，强化责任落实，确保各项工作高标准推进、高质量完成。加快政府职能转变，简化审批环节，提高行政效率。加强行政服务中心和电子政务建设，强化“一站式”服务，完善网上审批。加大治庸治懒力度，深化机关作风建设，形成勤政、务实、高效的良好政风。

建设廉洁型政府。坚持以人为本、执政为民，全面落实党风廉政建设责任制，加强行政监察和审计监督，强化对权力的监督和制约，树立公务员克己奉公、清正廉洁的良好形象。继续办好政风行风热线，坚决纠正行业不正之风。进一步增强宗旨意识、为民意识、服务意识，积极为民办好事实事。

　　各位代表，新的形势催人奋进，新的征程任重道远。让我们在省委省政府和市委的正确领导下，在市人大和市政协的监督支持下，以等不起的危机感，以坐不住的紧迫感，解放思想、抢抓机遇，开拓进取、奋起赶超，为加快转型升级、建设幸福潮州而不断努力！
